# Mapping motor and extra-motor gray and white matter changes in ALS: a comprehensive review of MRI insights

**DOI:** 10.1007/s00234-025-03629-7

**Published:** 2025-05-02

**Authors:** Valentina Virginia Iuzzolino, Alessandra Scaravilli, Guglielmo Carignani, Gianmaria Senerchia, Giuseppe Pontillo, Raffaele Dubbioso, Sirio Cocozza

**Affiliations:** 1 Department of Neurosciences, Reproductive Sciences and Odontostomatology, University of Naples Federico II, Naples, Italy; 2 Department of Advanced Biomedical Sciences, University of Naples Federico II, Naples, Italy

**Keywords:** Amyotrophic lateral sclerosis, Magnetic resonance imaging, Biomarker, Gray matter, White matter

## Abstract

Amyotrophic lateral sclerosis (ALS) is a progressive neurodegenerative disease primarily affecting motor neurons, yet with substantial clinical variability. Furthermore, beyond motor symptoms, ALS patients also show non-motor features, reflecting its classification as a multi-system disorder. The identification of reliable biomarkers is a critical challenge for improving diagnosis, tracking disease progression, and predicting patient outcomes. This review explores macro- and microstructural alterations in ALS, focusing on gray matter (GM) and white matter (WM) as observed through Magnetic Resonance Imaging (MRI). This approach synthesizes not only the expected involvement of motor areas but also highlights emerging evidence that these changes extend to extra-motor areas, such as the frontal and temporal lobes, underscoring the complex pathophysiology of ALS. The review emphasizes the potential of MRI as a non-invasive tool to provide new biomarkers by assessing both GM and WM integrity, a key advancement in ALS research. Additionally, it addresses existing discrepancies in findings and stresses the need for standardized imaging protocols. It also highlights the role of multi-modal MRI approaches in deepening our understanding of ALS pathology, emphasizing the importance of combining structural and diffusion MRI techniques to offer more comprehensive insights into ALS progression, ultimately advancing the potential for personalized treatment strategies and improving patient outcomes.

## Key points

**•** GM damage in ALS predominantly affects motor areas but also extends to non-motor regions such as the frontal, temporal, and parietal cortices.
**•** Longitudinal imaging studies suggest that GM atrophy and cortical thinning progress over time, correlating with disease activity and clinical stages.
**•** GM alterations in ALS can serve as markers to distinguish between ALS and healthy controls, as well as among groups with different onset-type.
**•** Diffusion MRI studies reveal significant microstructural involvement of GM in ALS, with decreased fractional anisotropy in motor areas correlating with disease severity and duration.
**•** While most studies show no significant WM macrostructural damage in ALS compared to healthy controls, some suggest a possible macrostructural involvement in the corticospinal tract and brainstem.

## Introduction

Amyotrophic lateral sclerosis (ALS) is a rapidly progressive and fatal neurodegenerative disease primarily affecting the motor system. It is characterized by progressive degeneration of upper motor neurons (UMN) in the cortex and lower motor neurons (LMN) in the brainstem and spinal cord, with a pathogenesis that remains partially unclear [1]. Clinically, ALS is highly heterogeneous, with variations in the site of onset, such as bulbar ALS (b-ALS) and limb ALS (l-ALS), as well as a differential involvement of UMN and LMN [2]. In recent years, in addition to the well-documented motor impairments, a non-motor dimension has been recognized, carrying significant prognostic implications [3]. This extra-motor involvement, indicative of the disease’s spread to non-motor brain regions, exhibits considerable variability and primarily encompasses cognitive [4, 5] and behavioural [6] disturbances that can progress to frontotemporal dementia, as well as autonomic dysfunction [7], sleep disturbances [8], sensory changes [9], or fatigue [10]. Identifying reliable biomarkers remains a critical yet partially unmet need in ALS research. Beyond aiding early diagnosis by distinguishing ALS from mimicking disorders, their identification could provide objective measures to monitor disease progression, enabling effective patient stratification. Additionally, these biomarkers may offer insights into disease prognosis, helping predict the course of ALS in individual patients. In the search for reliable biomarkers, neuroimaging techniques such as Magnetic Resonance Imaging (MRI) have emerged as valuable tools for investigating central nervous system (CNS) changes in ALS [11]. Being a non-invasive method for assessing both macro- and microstructural alterations associated with the disease, MRI provides valuable information about gray (GM) and white matter (WM) integrity, making it an appropriate candidate for identifying diverse and reliable biomarkers.

Given the numerous MRI studies on ALS published in recent years, this review aims to provide an updated overview of the current knowledge on macrostructural and microstructural changes affecting both GM and WM compartments observed in ALS using MRI. By synthesizing existing evidence, this review seeks to elucidate potential biomarkers for ALS and address eventual discrepancies in the literature.

### Gray matter macrostructural changes

The occurrence of GM changes in ALS is well-documented in the literature, with findings derived from different methods such as via volumetric analyses, voxel-based morphometry (VBM) studies or the assessments of cortical integrity, including cortical thickness (CT) and surface-based morphometry [12]. These GM alterations have been reported as possible useful markers to distinguish not only between ALS and healthy controls (HC), but also among groups with different onset-type [13, 14]. This differentiation has also been recently shown to be achievable using more complex classification methods, via the application of Machine Learning (ML) models [15]. However, the literature presents more conflicting findings regarding the sensitivity of these GM alterations and their relationship with the time of onset [16]. According to some authors, the reported reduction of CT only becomes detectable in later stages of the disease, with the observed different occurrence of this phenomenon that might depend on ALS onset-type [17]. Similarly, the literature reports conflicting results on the impact of pathology progression on cortical GM disruption in longitudinal studies [18–20]. However, it has been suggested that longitudinal imaging can reveal progressive changes in both atrophy and CT, which appear to be directly linked to active disease [17, 21, 22]. This finding is supported by studies on carriers of the C9orf72 variant, where these subjects showed no significant cortical thinning compared to HC in the asymptomatic phases of the disease but encountered a progressive cortical atrophy and reduction of CT during the symptomatic phases [23, 24]. This hypothesis of GM changes progressing over time alongside disease progression is also supported by a study that sub-classified ALS patients in early-stage (ALS-e) and late-stage (ALS-l) groups according to a 12 months of disease duration cut-off [25]. In this work, authors proved that the degree of GM atrophy in the ALS-e group was not as widespread as the one observed in ALS-l [25]. Nonetheless, it is noteworthy to mention that other groups failed to find significant longitudinal GM changes over time [20, 26–28], leaving the debate on the possible longitudinal changes affecting the GM still open.

**Fig. 1:**
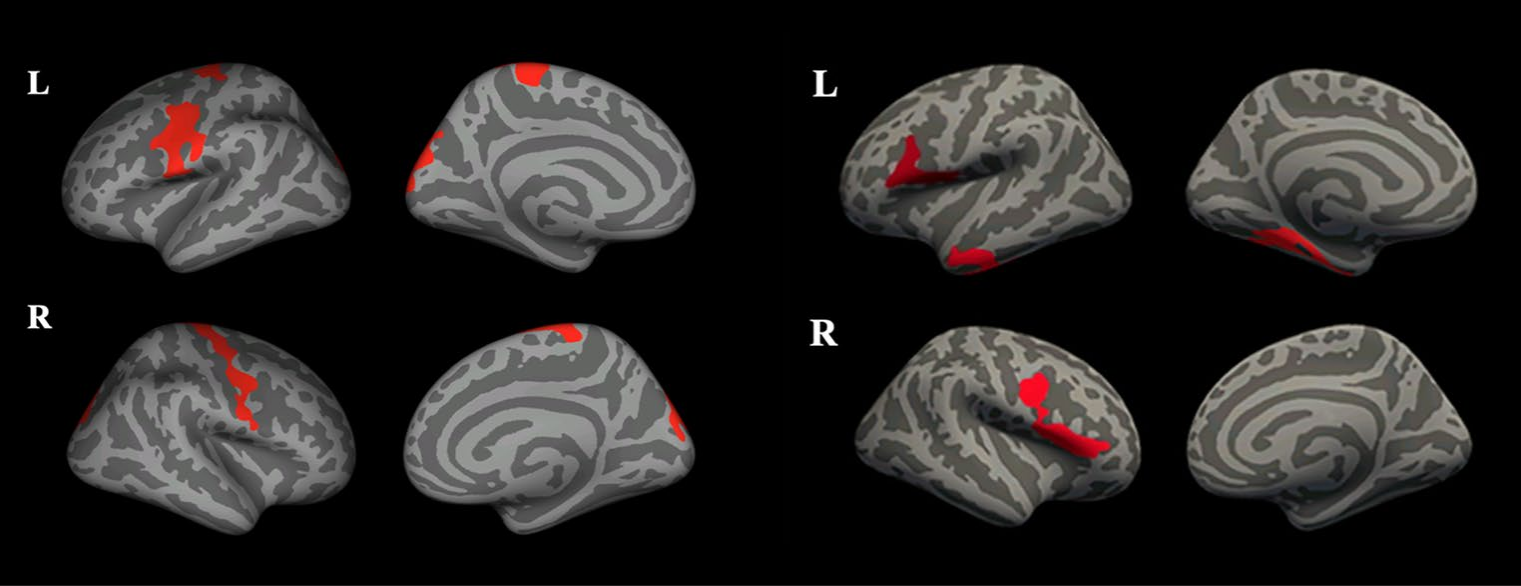
An example of brain regions showing a significant GM macrostructural involvement in ALS. An inflated brain surface image showing the occurrence of a significant cortical atrophy (images on the left) and cortical thinning (on the right) ALS patients. Modified from [34]. ALS: amyotrophic lateral sclerosis; GM: gray matter; L: left; R: right

Conversely, there is greater consensus in literature regarding the spatial distribution of GM damage in ALS, with significant and expected involvement of motor areas, particular of the precentral gyrus (PCG) [29–34] (Fig. 1). On the other hand, less univocal results have been reported with reference to the clinical correlates of these observed macrostructural changes of motor areas. In particular, some authors have found an association between GM volume of the PGC and clinical measures of ALS disability [33], while other failed to find significant associations [18, 35, 36]. Since the Revised ALS Functional Rating Scale (ALSFRS-R) reflects the involvement of both upper and lower motor neuron in the daily activities, it has been hypothesized that this could at least partly explain the absence of significant correlations between GM alterations and clinical variables [37]. Interestingly, some studies have suggested a possible correlation of ALSFRS-R sub-scores with alterations in distinct regions in the PCG, while the cortical volume, and especially its dorsal-lateral portion, was correlated with disability [25, 38–41]. This topological distinction in correlation with clinical scores was also proved when the different onset-type groups were investigated. In particular, b-ALS patients have been suggested to experience more pronounced GM atrophy in the bilateral head-face area, while l-ALS patients seems to exhibit a more marked volume loss in upper and lower limb areas, a finding consistent with their clinical onset-type [39, 42]. This distinct behavior between b-ALS and l-ALS patients was also observed in longitudinal comparisons, with a VBM analysis revealing that changes in GM concentrations were more pronounced in the b-ALS group, indicating a higher neurodegenerative burden in these patients [38]. Another piece of evidence for a possible topological distribution of GM damage in ALS comes from studies reporting that, in right-handed-onset ALS patients, disproportionate atrophy of the left motor cortical hand area occurred regardless of whether the onset of weakness was in a dominant or non-dominant limb [43]. These findings are supported by pathological studies that have demonstrated how the PCG is one of the regions with the highest concentration of cortical motor neurons [38, 42], reinforcing the concept of a “cortical focality” in ALS [44]. However, similar to some previously reported findings, it is important to note that other authors have shown a symmetric bilateral involvement of the PCG in these patients [45, 46], with some discrepancies potentially arising from the use of different methodologies or grouping strategies [47]. When patients were stratified based on their predominant involvement of either upper (UMN-ALS) or lower motor neuron (LMN-ALS), it was shown that UMN-ALS patients experience more pronounced cortical thinning of the PCG compared to those with an LMN-ALS onset [14]. Interestingly, no significant differences in terms of CT of the PCG have been reported when LMN-ALS patients and controls were compared [14]. In this context, patients with a classical ALS phenotype fall between UMN- and LMN-ALS groups, showing significant thinning of the PCG compared to controls, though not as pronounced as in UMS-ALS patients [14]. In the light of these results, a potential role of CT as an in-vivo marker of UMN impairment has been suggested. In clinical settings, LMN signs (e.g., weakness) are often easier to detect and quantify objectively compared to UMN signs, which may be masked in limbs with significant LMN involvement [19]. Supporting this possible role of the CT of the PCG as an MRI biomarker of UMN degeneration, this GM alteration has been proved to reliably predict UMN scores obtained at the clinical evaluation using standardized scales [32, 48–50].

A summary of the main findings is provided in the dedicated section of Table 1.

Although motor areas are predominantly affected in ALS, macrostructural damage of the GM extends beyond these regions, with significant involvement of several non-motor areas. Recent evidence has suggested significant GM damage in several regions, including the frontal, temporal and parietal cortices, both in terms of reduction of CT and GM volume [38, 51], with less frequent and less pronounced involvement of the occipital cortex and subcortical GM [38, 40, 41, 43, 52–55]. This macrostructural GM involvement of non-motor areas has been also linked to different clinical features and ALS onset-types. For instance, rapid disease progression has been associated with more pronounced cortical thinning of the temporal lobe and of the sensorimotor cortex [26, 27, 56, 57], while a reduction in GM of the bilateral frontal cortex seems to correlate with alterations in executive functions [36]. In bulbar-onset patients, macrostructural GM damage is relatively widespread, affecting the bilateral frontotemporal, superior temporal and supramarginal gyri [38], as well as regions of the Speech Network [58], a finding in line with the observation that these patients are more likely to develop cognitive, behavioral and speech impairments [59]. However, the exact distribution of extra-motor areas involvement varies considerably across different studies [38, 60–63].

Of particular interest are the emerging findings regarding cerebellar involvement in ALS. It is now well established that the cerebellum plays a role beyond traditional motor control, encompassing cognitive functions such as memory, emotion, and language [64]. In this context, ALS patients, particularly those with cognitive impairment, have shown GM alterations in the bilateral cerebellar cortex [17, 38, 52, 53, 65–68]. An evaluation of CT profiles of this region has revealed a different preferential involvement of specific areas in variant carriers ALS patients or in subjects with a cognitive disruption, with only some cerebellar regions (i.e., lobules I–V of the anterior lobe in patients with sporadic ALS or posterior lobe and vermis in C9orf72 mutation carriers) apparently spared by this thinning [52, 69, 70]. Interestingly, an increase in cerebellar CT has been observed in ALS patients compared to controls in a few scattered cortical areas, a finding interpreted as an adaptive response to compensate for GM atrophy in the PCG [71]. Although some studies have failed to demonstrate significant differences in the global, lobular, or cerebellar dentate nucleus GM volume in ALS patients [72], evidence suggests that these cerebellar alterations progress over time, as shown in longitudinal evaluation [17].

### Gray matter microstructural changes

Along with volume loss and cortical thinning, ALS patients also experience significant microstructural involvement of the GM. Although different imaging techniques can evaluate brain microstructure, diffusion MRI (dMRI) plays an unquestionable central role. Using Diffusion Tensor Imaging (DTI) models, a decrease in fractional anisotropy (FA) -a sensitive, although non-specific, index of microstructural integrity- has been reported in motor areas such as the supplementary motor area, showing significant correlations with disease severity and duration [73]. A similar reduction in FA values has been described at the level of subcortical GM structures, particularly in the caudate nucleus [74] and thalamus [74, 75]. Despite the well documented and previously described macrostructural alterations of the PCG, evidence regarding its microstructural changes remains inconsistent. One study reported a reduction in FA within the PCG in ALS patient, as well as in the supplementary motor cortex, while other diffusivity metrics appeared increased in the remaining portions of the motor system [76]. Moreover, FA in the PCG was found to be significantly reduced in longitudinal evaluations [76]. This observed low consistency of evidence on microstructural alterations of the PCG might be at least in part ascribable to technical limitations of the DTI model, which is not specifically designed for the detection of GM changes due to directionality of diffusion traditionally being attributed to (myelinated) axons. Interestingly, while clinical variables such as disease duration and ALSFRS-R correlate with FA values in the PCG, changes in other diffusion measures do not appear to be significantly associated with clinical parameters [76]. This finding warrants further investigation, as it remains unclear whether the observed microstructural alterations are entirely attributable to GM changes or may reflect juxtacortical WM damage. Different studies dividing the corticospinal tract (CST) into different segments showed the occurrence of changes affecting the WM immediately adjacent to the PCG [77–79]. Interestingly, significant alterations in another dMRI variable, namely the Mean Diffusivity (MD), but not in FA, were observed in both cortical (i.e., frontal cortex and hippocampus) and subcortical (i.e., caudate, thalamus, and amygdala) GM structures in ALS patients [80]. A possible explanation for this discrepancy may lie in the inherently higher sensitivity of FA in detecting changes occurring in WM microstructure compared to GM [80]. Overcoming some of the limitations of DTI, advanced models such as Diffusion Kurtosis Imaging (DKI) can offer a more detailed characterization of GM microstructure by accounting for the non-Gaussian properties of water diffusion in complex brain tissues [81]. Interestingly, some DKI-derived measures (including the apparent kurtosis coefficient and axial, radial and mean kurtosis) outperformed conventional DTI measures in distinguishing ALS from controls [82], with decreased mean kurtosis and radial kurtosis values that have been reported in bilateral motor areas and the anterior cingulate gyrus in ALS patients [83].

A detailed summary of the main results is presented in the corresponding section of Table 1.

**Fig. 2:**
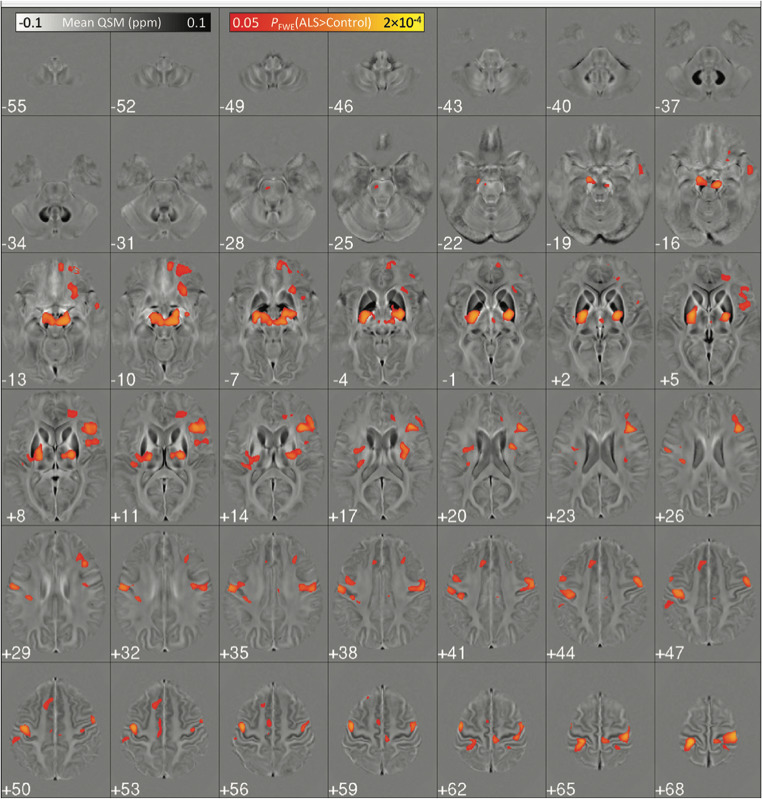
Extension of GM microstructural involvement in ALS. Results of a voxel-based analysis of QSM data showing areas of increased QSM values in ALS patients compared to controls, suggesting the degree of GM microstructural involvement occurring in these patients. Reprinted with permissions from [85]. ALS: amyotrophic lateral sclerosis; GM: gray matter; QSM: quantitative susceptibility mapping

Nonetheless, diffusion MRI is not the only technique available for evaluating brain microstructure. This is particularly relevant for GM, where Quantitative Susceptibility Mapping (QSM) plays an important role as a technique sensitive to local susceptibility changes, such as those induced by iron [84]. Several QSM studies have investigated susceptibility changes in the PCG, revealing increased tissue iron accumulation in ALS [85]. Furthermore, this increase in tissue iron load in ALS appears to extend beyond the PCG to deep gray matter nuclei, including the substantia nigra, globus pallidus, red nucleus, and putamen [85] (Fig. 2). Moreover, iron accumulation in the PCG appears to be more pronounced in UMN-predominant ALS phenotype compared to LMN-ALS [86]. Integrating QSM with supplementary quantitative measures, such as cerebral blood flow and GM volumetry of the motor cortex, offers a promising approach for effectively distinguishing UMN-predominant ALS from Primary Lateral Sclerosis (PLS) [87]. Furthermore, alterations in QSM values in the motor cortex of ALS patients have been associated with disease-specific functional disability and disease duration [87]. QSM analyses in the motor cortex have demonstrated the ability to differentiate ALS patients and HC [85], as well as between ALS and ALS mimics [88].

### White matter macrostructural changes

Although most of the current literature reports no significant differences in WM macrostructural damage between ALS patients and to HC [15, 89–91], and generally a lack of correlation with clinical scores, some studies have suggested a possible WM macrostructural involvement in ALS, with a degree of correlation with motor symptoms [38, 66]. In particular, as might be expected, macrostructural changes affecting the CST have been reported in literature, with a significant reduction of its volume compared to HC [92–94]. This volume loss has been suggested to be more prominent at the level of the posterior limb of the internal capsule [85, 92], although this finding has not been consistently reported across all studies [93]. Furthermore, WM macrostructural changes have been reported also in other major WM bundles, as well as brain areas where different WM fibers are localized, such as the brainstem. For instance, one study has reported a decrease in brainstem WM volume across all its components in PLS patients [75]. On the other hand, while the entire brainstem volume is reduced in ALS patients compared to controls, a more prominent atrophy has been reported in the pons and medulla compared to the one observed in the midbrain [95]. However, this finding is not consistently reported across studies, with a study suggesting a significant decrease in midbrain volume, particularly in the region corresponding to the pyramidal tract within the cerebral peduncle [96]. In line with the natural history of the disease, these volumetric alterations appear to become more pronounced over time, as demonstrated in a longitudinal study conducted over a four-month observation period [95]. Furthermore, a possible difference in brainstem macrostructure between ALS and PLS patients has been suggested, as shape deformations have been reported in the pons and medulla (particularly affecting their ventral portion) in ALS patients [97], a finding not observed in PLS patients [95]. Additionally, no cerebellar white matter volume reductions were observed [69].

Regarding the possible clinical relevance and different biological counterparts of these WM macrostructural changes, it has been reported that l-ALS patients may exhibit more pronounced WM volume loss in motor-related areas, whereas b-ALS subjects appear to have more widespread WM involvement, including also extra-motor regions such as the supplementary motor area, the superior frontal or inferior temporal areas [66]. However, when compared to HC, WM analysis in both b-ALS and l-ALS patients also revealed decreased density within the associated fiber tracts of the bifrontal, bitemporal and biparietal lobes [38]. Finally, patients with cognitive impairment showed decreased WM volume in both superior longitudinal fasciculi compared to patients without cognitive impairment. On the other hand, no differences were observed between patients with behavioral impairment and those without this feature [38].

**Fig. 3:**
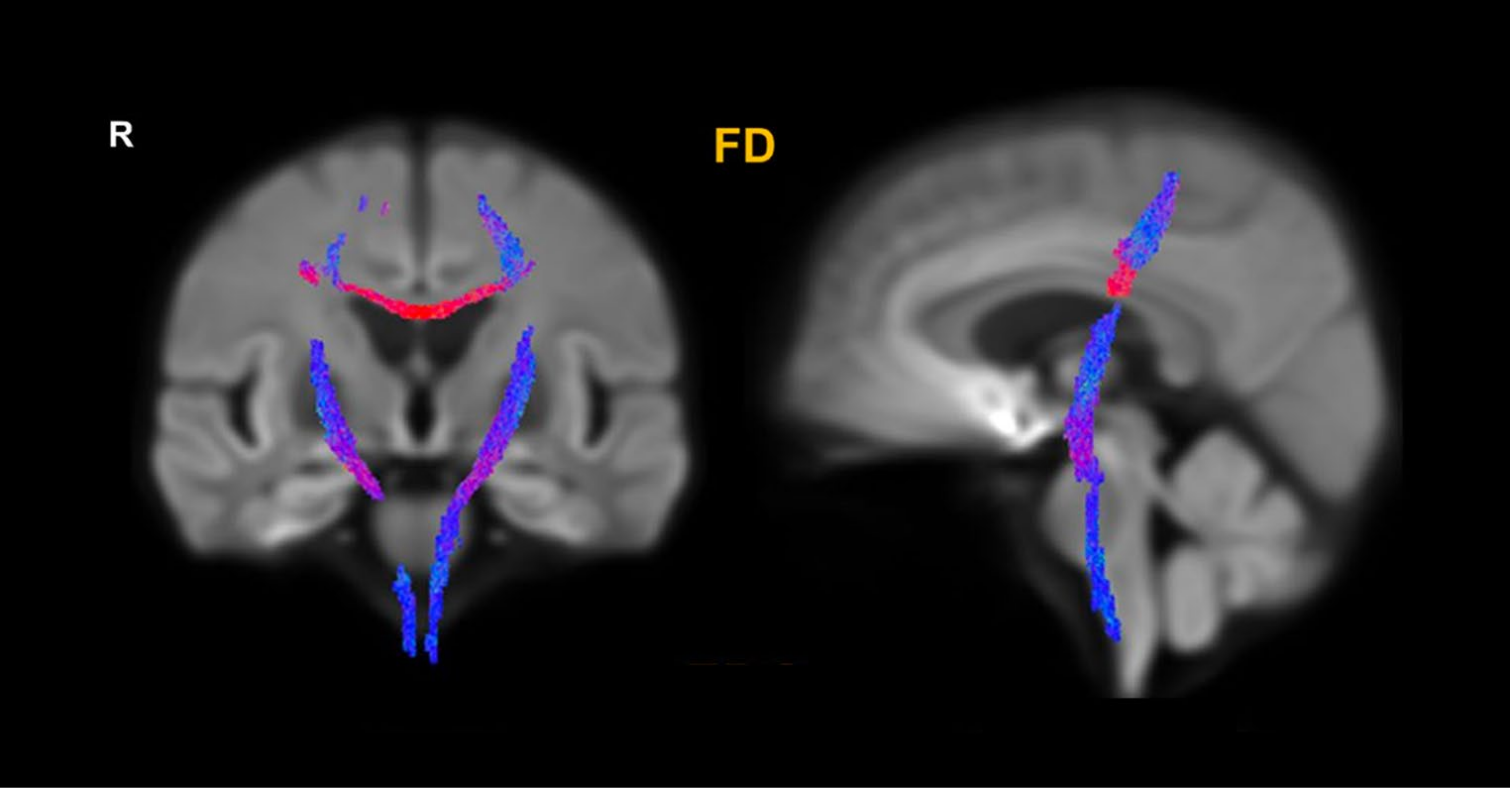
An example of WM macrostructural involvement in ALS. Results of a fixel-based analysis of dMRI data showing a significantly decreased fiber density in the CSTs and the CC in ALS. Reprinted with permissions from [127]. ALS: amyotrophic lateral sclerosis; WM: white matter; dMRI: diffusion magnetic resonance imaging; CST: corticospinal tract; CC: corpus callosum

A graphical representation of the main WM macrostructural findings in ALS can be found in Fig. 3.

Table 1 outlines the main findings in its designated section.

### White matter microstructural changes

Microstructural damage of the WM in ALS is a well-documented phenomenon, primarily investigated through dMRI studies. In particular, WM damage in ALS predominantly affects the CST and other motor-related areas, including the corona radiata and the posterior limb of the internal capsule, as well as regions such as the midbrain, medulla, and middle corpus callosum [83, 98–101]. In particular, several studies using DTI have reported decreased FA values in the CST [102–106], corpus callosum, cingulum [60, 101, 107–109], and frontal and pre-frontal areas [110]. These FA changes are often accompanied by an increase in Radial Diffusivity (RD) [111, 112], which can be used as a marker of demyelination [113, 114]. Nonetheless, the reduction in FA within the CST remains the most consistent DTI finding in ALS and is significantly correlated with both the rate of disease progression [105, 115, 116] and the ALSFRS-R [18, 75]. Significant abnormalities of the brainstem portion of the CST are also reported in ALS, especially in patients with lower performance in bulbar and upper limb function, indicated by lower FA and increased RD values [111, 117–119]. Another consistently affected structure in ALS is the corpus callosum, which shows decreased FA and increased MD values in DTI studies [29, 120], reflecting an interhemispheric disease spread via callosal fibers [45, 105, 108, 121–125]. Segmental alterations of FA in callosal segments I, II, and III appear to be a common MRI feature across all ALS phenotypes, with segment III being particularly affected. Interestingly, this callosal portion, corresponding to the motor segment of corpus callosum [126, 127], is notably affected in both sporadic ALS [128] and familial ALS harboring C9orf72 expansion [129].

Possibly due to the propagation of WM changes from motor to extra-motor areas as the disease progresses [130], ALS patients also experience microstructural involvement of numerous extra motor areas, clinically manifesting as alteration in executive functions and behavioral impairments [131–134]. In particular, different DTI studies in ALS patients with cognitive impairment have shown a reduction in FA values of the WM adjacent to the cingulum, the superior longitudinal fasciculus, the uncinate fasciculus [131, 135–137] and parahippocampal gyrus [126, 138]. The involvement of the cingulum has often been reported in relation to cognitive impairment [131, 135–137, 139], while decreased parietal WM integrity has been specifically reported in patients with behavioral impairments [139, 140]. Microstructural changes in the cortico-ponto-cerebellar system have also been reported [97, 123] and seem to be related to memory disfunction [141] in ALS patients. Furthermore, WM damage in prefrontal areas, along with a reduction in FA values in frontal association fibers, appears to be related to alteration in language and cognitive performance [142], with significantly worse performance in all Edinburgh Cognitive and Behavioral ALS Screen (ECAS) domains [142]. Similarly, involvement of the inferior longitudinal fasciculus and inferior frontal occipital fasciculus has been reported in association with lower ECAS verbal fluency scores [143], while callosal involvement seems to be associated with lower ECAS memory scores, likely due to damage to thalamocortical circuits [21, 80, 143, 144]. Interestingly, cerebellar WM involvement has been reported in patients with ALS and frontotemporal dementia (ALS-FTD) [145]. In particular, in C9orf72 expansion carriers [146] a widespread, symmetric reduction in FA of cerebellar WM has been described [70], while in C9orf72-positive patients, involvement of the superior peduncle has been reported, characterized by a reduction in FA and an increase in RD values [70].

According to a recent study, the pattern of WM microstructural damage in ALS evaluated via DTI can describe three distinct MRI phenotypes, each associated with a different clinical profile [147]. Specifically, a pure motor cluster is defined by lower FA values in the CST and surrounding WM tracts, representing the first profile. A frontotemporal cluster shows preferential frontotemporal WM degeneration and an impaired WM network connecting orbitofrontal and anterior temporal regions. The third phenotype, namely the cingulate-parietal-temporal, involves tracts connected with the posterior cingulate cortex and the superior parietal cortex [147].

Finally, it is noteworthy that additional advanced diffusion models, such as DKI and neurite orientation and dispersion density imaging, can provide an even more accurate characterization of WM microstructural damage in ALS. This is reflected by evidence of decreased mean and radial kurtosis, and neurite density index, respectively, in areas known to be affected, such as the CST, PCG WM, middle portion of the corpus callosum, and frontotemporal-related tracts [83, 99, 148, 149]. All these microstructural findings align with neuropathological studies showing the presence of axonal loss and demyelination in the CST and spinal cord [150–152] along with callosal degeneration [153].

**Fig. 4:**
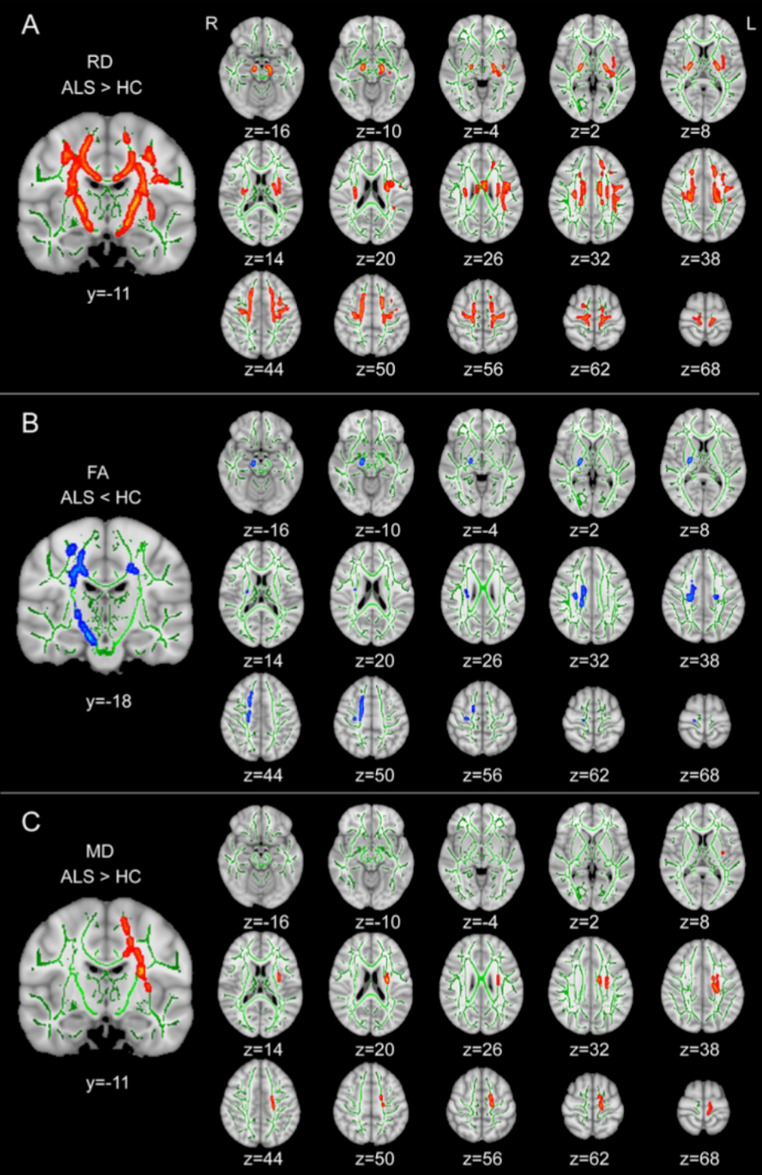
Degree of WM microstructural involvement in ALS. Results of a TBSS analysis showing the degree of WM microstructural changes occurring in in ALS patients, with altered RD, FA and MD values across the whole brain (WM skeleton in green). Regions in yellow/red scale indicate a significant increase in RD (upper panel, **A**) and MD (lower panel, **C**), while blue-light blue scale indicate significant decrease in FA values (middle panel, **B**). Reprinted with permissions from [112]. ALS: amyotrophic lateral sclerosis; WM: white matter; dMRI: diffusion magnetic resonance imaging; TBSS: tract-based spatial statistics; RD: radial diffusivity FA: fractional anisotropy; MD: mean diffusivity

A graphical representation of the main WM microstructural findings in ALS can be found in Fig. 4.

Table 1 includes a dedicated section summarizing the main findings.

**Table 1:** Summary of the main macro- and microstructural MRI changes in ALS

Gray Matter			White Matter	
	*Motor areas*	*Extra-motor areas*	*Motor areas*	*Extra-motor areas*
**Macrostructure**	Significant and expected involvement, particularly of the precentral gyrus, which is more pronounced in bulbar-onset ALS Reported progression over time linked to active disease, with some conflicting results about longitudinal changes over time	Significant involvement, of the frontal, temporal and parietal cortices, and less frequently of the occipital cortex and the subcortical structures Widespread changes in bulbar-onset patients, mainly involving regions of the Speech Network Cerebellar involvement reported in subject with cognitive impairment	Decreased volume of CST, more prominent at the level of the posterior limb of internal capsule, also more pronounced over time Occurrence of a pronounced involvement in limb-onset ALS, while it appears to be more widespread in bulbar-onset patients	Decreased volume of brainstem and corpus callosum, which becomes more pronounced over time More pronounced and widespread in bulbar-onset patients Decreased volume in superior longitudinal fasciculi in patients with cognitive impairment
**Microstructure**	Decreased FA values in the supplementary motor area, showing significant correlations with disease severity and duration Low consistency of the findings affecting the precentral gyrus Increased tissue iron accumulation in the precentral gyrus, more pronounced in upper motor neuron predominant phenotypes Alterations in QSM values associated with disease-specific functional disability and disease duration	Decreases FA values in subcortical gray matter structures, particularly in the caudate nucleus and thalamus Significant MD alterations in different cortical (i.e., frontal cortex and hippocampus) and subcortical (i.e., caudate, thalamus, and amygdala) structures Increase in tissue iron load in deep gray matter nuclei, including the substantia nigra, globus pallidus, red nucleus, and putamen	A significant reduction in FA values within the CST is the most consistent finding in ALS, significantly correlating with clinical scores and disease progression Abnormalities of the brainstem portion of the CST, especially in patients with lower performance in bulbar and upper limb function Segment III (corresponding to the motor portion) of the corpus callosum is particularly affected	Decreased FA and increased MD values in corpus callosum, reflecting a possible interhemispheric disease spread via callosal fibers Reduction in FA adjacent to the cingulum, the superior longitudinal fasciculus, the uncinate fasciculus and parahippocampal gyrus in patients with cognitive impairment Decreased parietal and prefrontal and frontal association fibers integrity in patients with behavioral impairment and with alteration in language and cognitive performance, respectively

ALS = Amyotrophic Lateral Sclerosis; CST = corticospinal tract; FA = Fractional Anisotropy; MD = Mean Diffusivity; QSM = Quantitative Susceptibility Mapping

## Conclusions

In conclusion, this review underscores the critical role of MRI in investigating both GM and WM alterations in ALS. Although numerous studies have demonstrated the occurrence of GM macrostructural changes predominantly affecting the motor cortex, this involvement also occurs in extra-motor regions such as the frontal and temporal lobes. These findings reinforce the understanding that ALS is not merely a motor disease but also encompasses different non-motor areas that contribute to the wide clinical heterogeneity observed in these patients. In addition to GM changes, the use of dMRI techniques has highlighted the occurrence of significant microstructural damage in the CST and other motor-related pathways, in line with the clinical signs of motor dysfunction in ALS.

While MRI provides a non-invasive method to assess macro- and microstructural alterations in ALS, several challenges remain, including discrepancies in some findings and the need for standardized imaging protocols. It is noteworthy that part of the discrepancies observed between studies may be attributable to methodological issues in data acquisition (i.e. the use of different MRI scan and protocol) and analysis (e.g., the use of different software or the co-variates added in the models [154, 155]), as well as the use of different scales and/or weaknesses within the clinical measures used [156, 157]. As demonstrated in other fields [158], a standardization framework, including minimal requirements for basic imaging while still allowing for the incorporation of higher-level imaging techniques necessary for advanced models, is a challenging but achievable task. By ensuring greater comparability across international cohorts, this dual approach would support both foundational consistency and the flexibility to include cutting-edge methods where available, achieving a balance between clinical feasibility and the advancements derived from the research field.

Future research should focus on longitudinal studies to track disease progression and validate these biomarkers’ predictive power. Furthermore, integrating multi-modal imaging approaches by combining structural, diffusion, and functional MRI could offer a more comprehensive understanding of ALS pathology. Moreover, integrating neuroimaging with other biomarkers, such as fluid analyses and genetic testing, can provide a holistic understanding of ALS. Lastly, the application of ML models for MRI data analysis promises to refine diagnosis and prognosis, ultimately leading to improved patient outcomes. Effectively translating these findings into clinical practice—particularly through enhanced patient stratification and personalized treatment strategies—is essential for optimizing patient care.

## Data Availability

No datasets were generated or analysed during the current study.
